# Fabrication of Transparent Mg(OH)_2_ Thin Films by Drop-Dry Deposition

**DOI:** 10.3390/ma14040724

**Published:** 2021-02-04

**Authors:** Tong Li, Masaya Ichimura

**Affiliations:** Department of Electrical and Mechanical Engineering, Nagoya Institute of Technology, Gokiso, Showa, Nagoya 466-8555, Japan; 31413210@stn.nitech.ac.jp

**Keywords:** Mg(OH)_2_, drop-dry deposition, transparent conductive thin film

## Abstract

Magnesium hydroxide (Mg(OH)_2_) thin films were deposited by the drop-dry deposition (DDD) method using an aqueous solution containing Mg(NO_3_)_2_ and NaOH. DDD was performed by dropping the solution on a substrate, heating-drying, and rinsing in water. Effects of different deposition conditions on the surface morphology and optical properties of Mg(OH)_2_ thin films were researched. Films with a thickness of 1−2 μm were successfully deposited, and the Raman peaks of Mg(OH)_2_ were observed for them. Their transmittance in the visible range was 95% or more, and the bandgap was about 5.8 eV. It was found that the thin films have resistivity of the order of 10^5^ Ωcm. Thus, the transparent and semiconducting Mg(OH)_2_ thin films were successfully prepared by DDD.

## 1. Introduction

In recent years, transparent conductive materials (TCMs) have been widely used as electrode materials for transparent electronics devices such as liquid crystal displays and touch panels, and also for thin film solar cells. Use of liquid crystal displays is rapidly spreading, and the demand for economical solar cells is increasing because of the climate change crisis. Thus, there is an increasing need for transparent electrodes. In addition, TCMs have begun to be used as semiconductors in devices such as thin film transistors (TFTs) [1,2,3]. Many oxides are transparent because they have a large bandgap, and transparent conductive oxides (TCOs) have been used for transparent electrodes. Among TCOs, indium tin oxide (ITO) has a low resistivity and a high transmittance and therefore is widely used at present. However, its constituent element indium is a rare metal, not abundant in the Earth’s crust. Thus, there is a need for a material that can replace ITO, considering the possibility of resource depletion in the future. On the other hand, the most promising TCM for transparent TFTs is InGaZnO (IGZO) [4], but extensive research has also been carried out to develop TFT based on other materials such as ZnO [5] and SnO_x_ [6].

This study focused on magnesium hydroxide (Mg(OH)_2_). Mg(OH)_2_ has a bandgap of about 5.7 eV [7], is transparent to visible light, and has been generally used in the chemistry field as an antacid or flame retardant. The constituent element Mg is an abundant substance with the eighth largest Clarke number. Impurity doping in Mg(OH)_2_ was investigated by first-principles calculation, and it was predicted that Mg(OH)_2_ can have both n and p-type conductivity by proper impurity doping [8]. Several research groups actually fabricated solar cells with a Mg(OH)_2_ layer inserted to enhance output voltage [9,10,11]. In addition, by reacting a carbon-doped Mg film with moisture, conductive carbon-doped Mg(OH)_2_ with resistivity of the order of 10^−2^ or 10^−3^ Ωcm was prepared [12,13,14,15]. Thus, although Mg(OH)_2_ has been traditionally regarded as an insulator, it can also be used as a semiconductor or conductor in electronics applications.

Many fabrication methods of Mg(OH)_2_ have been reported, including the hydrothermal synthesis method [16], chemical precipitation method [17,18,19,20,21,22,23], sol-gel technique [24], microwave-assisted synthesis [25], surfactant-mediated growth method [26], electrochemical deposition (ECD) method [27,28], etc. It was reported that Cu-doped Mg(OH)_2_ fabricated by ECD is semiconducting [29].

**Figure 1:**
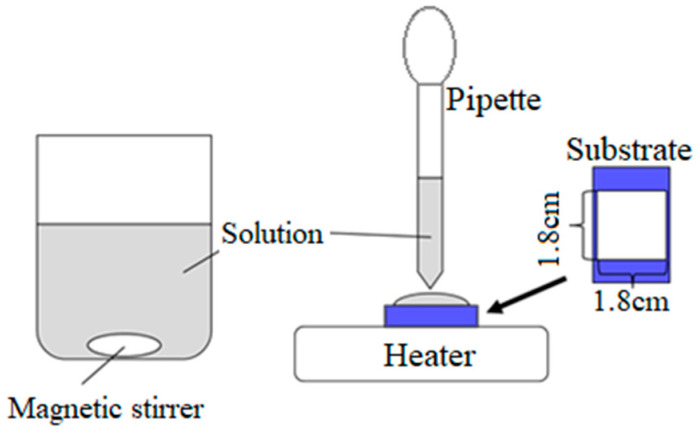
Apparatus of the drop-dry deposition method.

In this paper, we report the fabrication of Mg(OH)_2_ films by a simple technique: drop-dry deposition (DDD). DDD is a method of depositing a thin film by dropping and drying a solution on the substrate as shown in Figure 1. It uses a heating plate only and does not need other apparatuses, e.g., vacuum chamber, electric power supply, or light source. Thus, the apparatus required by DDD is simple and easy to use, and therefore DDD is advantageous for deposition of a thin film over a large area at a low cost. As shown below, the deposited Mg(OH)_2_ films are highly transparent and semiconducting.

## 2. Experiments

For the Mg(OH)_2_ preparation, Mg(NO_3_)_2_ and NaOH were dissolved in pure water. After mixing, Mg(OH)_2_ was spontaneously synthesized by the reaction:

Mg^2+^ + 2OH^−^ = Mg(OH)_2_, (1)

Without stirring, Mg(OH)_2_ particles are physically entangled to form aggregates of a specific size. However, with stirring, the entanglement is loosened [30], and a uniform, slightly hazy solution was obtained.

The substrate was alkali-free glass. Several samples were also prepared on quartz substrates for optical characterization. Before the thin film deposition, the substrate was degreased and washed with acetone and pure water, and the deposition area was limited to 1.8 × 1.8 cm^2^ by masking. The deposition solution was dropped on the substrate using a pipette. Then, the substrate was heated at 60 °C using a heater plate until the water was evaporated completely. The time required for drying was about 10 min when 0.2 mL was dropped. Then, the substrate was rinsed with pure water and blown by a nitrogen gas. During the drying process, Mg(OH)_2_, which has low solubility in water, first precipitates and is deposited on the substrate. After that, other solutes, having higher solubility, precipitate on the film, and then they are washed away in the subsequent rinsing process. The steps of the solution dropping and drying were repeated several times to deposit the thin film.

The deposition was carried out under the following conditions:

- Number of drop-dry cycles: 5, 10 (Mg(NO_3_)_2_: 25 mM, NaOH: 50 mM).
- Mg(NO_3_)_2_ concentrations: 10, 20, 30, 40, 50 mM (NaOH: 50 mM, cycles: 5).
- NaOH concentrations: 25, 50, 100 mM (Mg(NO_3_)_2_: 25 mM, cycles: 5).

Film thickness and surface roughness were measured by an Accretech Surfcom-1400D profilometer. Optical transmittance measurement was performed using a Jasco V-570 UV/VIS/NIR spectrometer. Auger electron spectroscopy (AES) data and scanning electron microscope (SEM) images were obtained using a JEOL JAMP-9500F field emission microprobe (JEOL LTD., Akishima, Japan) at a probe voltage of 10 keV. Raman spectroscopy measurement was performed with excitation laser wavelength of 532 nm using a Jasco NRS-3300 Raman spectroscope (JASCO Corporation, Tokyo, Japan). X-ray diffraction (XRD) experiments were performed with a SmartLab X-ray diffractometer (Rigaku) using a Cu Kα radiation source. For the electrical characterization, indium inter-digit electrodes were fabricated by vacuum evaporation on the thin film prepared on the alkali-free glass substrate, and then current-voltage (I-V) measurement was performed.

## 3. Results and Discussion

### 3.1. Deposition Conditions

Thickness was measured for the thin films prepared under the various deposition conditions noted above. When the deposition cycle was repeated five times, the film thickness was about 2.0 μm. When the drop-dry (DD) cycle number exceeded seven, the film began to become hazy or porous. Then, the apparent thickness increased steeply, and the film became mechanically weak. When the cycle number was increased to 10, the average thickness increased to about 10 μm, with nonuniformity much enhanced. The thickness was more than 10 μm in some parts, but some parts of the film fell off the substrate. With increasing Mg(NO_3_)_2_ concentration (10–40 mM), the film thickness increased from 0.7 μm to 3.0 μm, and its nonuniformity became larger. At 50 mM, a part of the thin film was broken and fell off the substrate. With increasing NaOH concentration (10–100 mM), the film thickness increased from 0.8 μm to 2.2 μm, and the uniformity did not change significantly.

**Figure 2:**
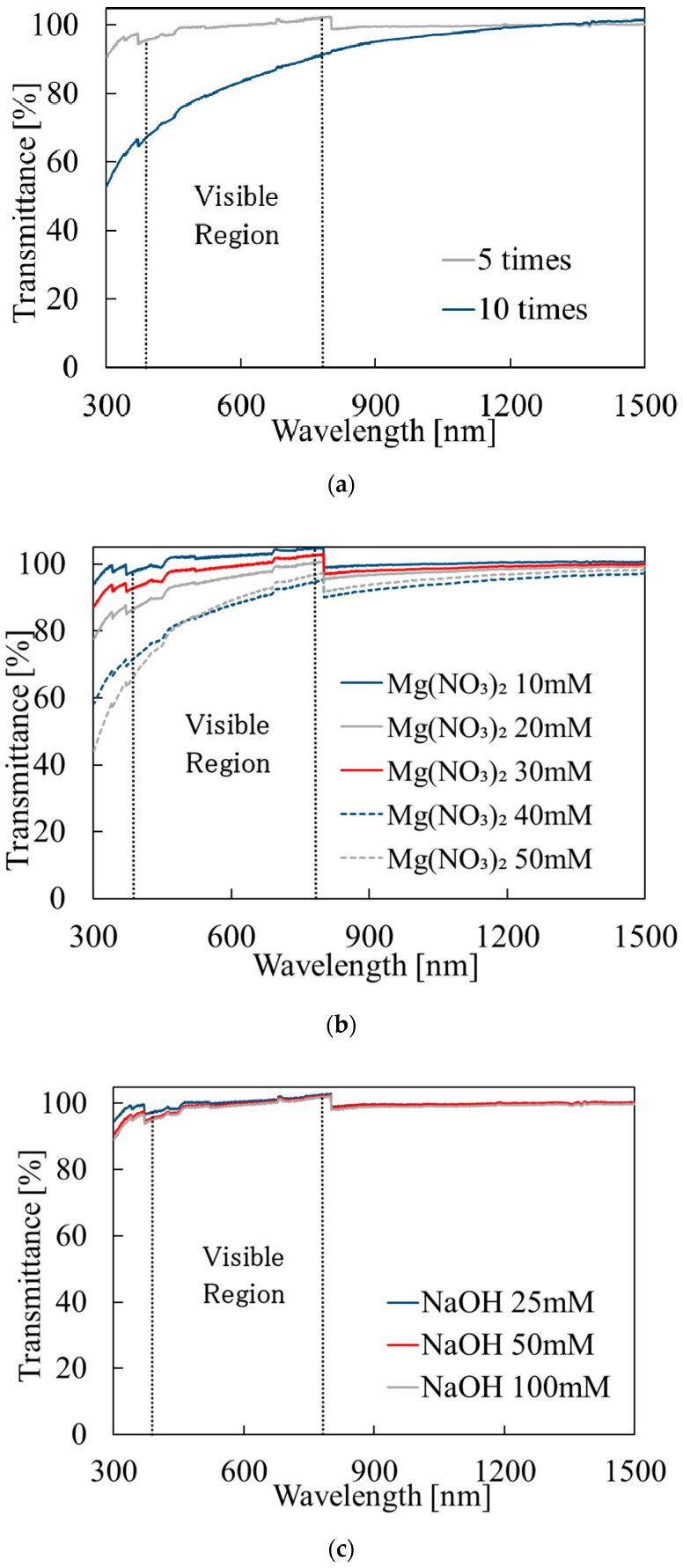
Optical transmittance under each deposition condition: (**a**) change in the number of depositions; (**b**) change in Mg(NO_3_)_2_ concentration; (**c**) change in NaOH concentration. (The steps near 800 nm were caused by sensor changes in the spectrophotometer, and we failed to calibrate the signal level.)

Figure 2 shows the optical transmittance of the thin films prepared under different deposition conditions. The data plotted in Figure 2 were obtained by dividing the transmittance of the sample by the reference data taken for the glass substrate without any deposit on it. As shown in Figure 2a, the sample deposited with 5 cycles showed transmittance of 95% or more in the visible region, but the sample deposited with 10 cycles had lower transmittance (65–90%) in the visible region: The 10-cycle sample was hazy. Figure 2b shows the influence of Mg(NO_3_)_2_ concentration. For Mg(NO_3_)_2_ concentrations ≤ 30 mM, the samples showed high transmittance (>90%) in the visible region. However, when the concentration of Mg(NO_3_)_2_ was 40 mM or more, the transmittance was significantly decreased, 65–95% in the visible region. Although the transmittance tended to decrease with increasing Mg(NO_3_)_2_ concentration, the transmittance was higher for 30 mM than for 20 mM. This could be because of nonuniformity in surface roughness: the data for 20 mM are considered to be affected by local surface roughness. Thus, the reproducibility is not good enough to discuss a difference of a few percentage points in transmittance. Figure 2c shows the effects of NaOH concentration. The samples deposited with NaOH concentrations of 25, 50, and 100 mM showed transmittance of 95% or more in the visible region, and thus the effects of NaOH concentration are not significant. The transmittance of some samples appeared to exceed 100% because of lower reflectance of Mg(OH)_2_ than glass. As noted above, the glass substrate was used as a reference. Since Mg(OH)_2_ has a larger bandgap, the refractive index of Mg(OH)_2_ is expected to be lower than that of glass. Thus, for the glass substrate with Mg(OH)_2_ on it, the reflectance is lower and the transmitted light power can be higher than for that of the bare glass substrate. Then, the apparent transmittance value exceeds 100%. It should be noted that the transmittance did not exceed 100% when a quartz substrate was used, as shown below. This is because quartz has a larger bandgap and lower refractive index than Mg(OH)_2_.

**Figure 3:**
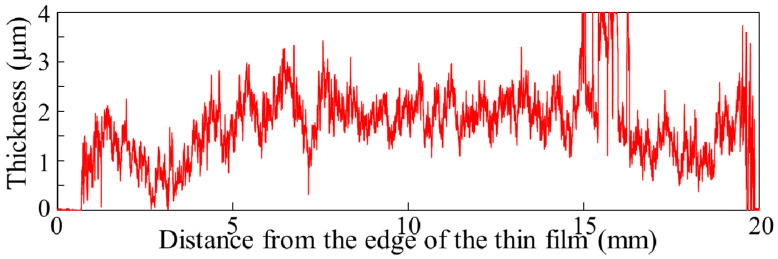
Surface roughness measurement results of the Mg(OH)_2_ film.

Considering both the thickness and transmission results, we can conclude that when the thickness exceeds 3 μm, the roughness and nonuniformity become so large that the transmittance in the visible range is significantly low. In the following characterization, we adopted the deposition conditions: 25 mM Mg(NO_3_)_2_, 50 mM NaOH, and 5 deposition cycles. Under this condition, the film thickness was about 2 μm, and the transmittance was 95% or more in the visible range. The thickness profile measurement results are shown in Figure 3. We repeated deposition under these conditions, and the film thickness and transmittance were reproduced with deviations of about 5%.

### 3.2. Characterization of the Films

**Figure 4:**
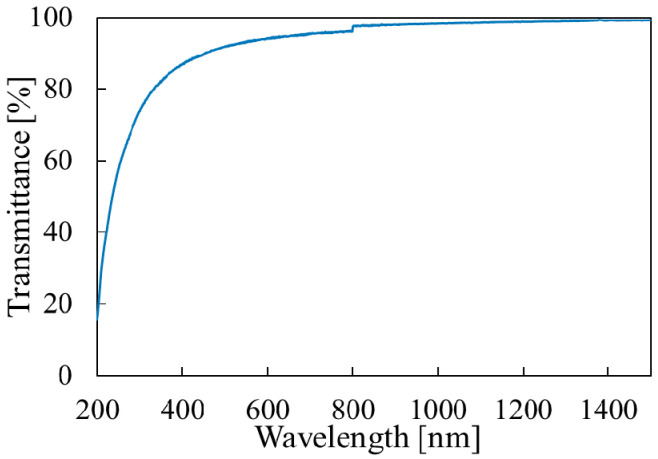
Optical transmittance measurement results of the Mg(OH)_2_ film.

**Figure 5:**
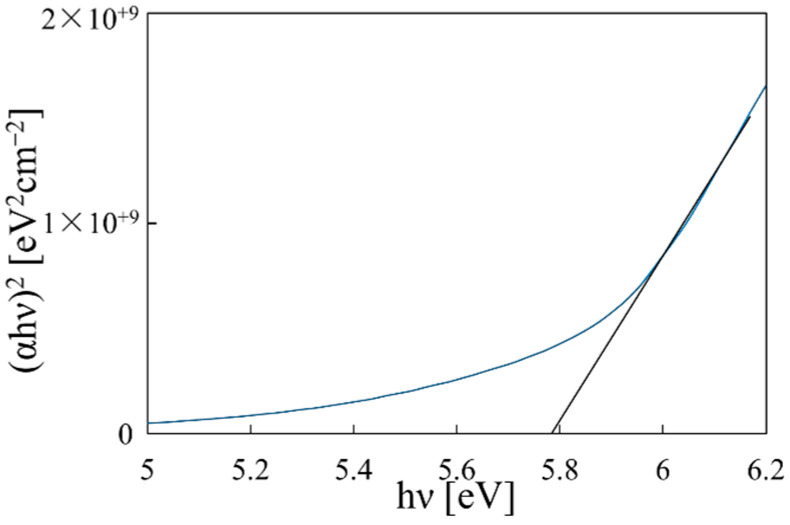
Bandgap evaluation of the Mg(OH)_2_ film.

To evaluate the bandgap, we deposited the film on a quartz substrate, which does not absorb UV light (>200 nm). The transmittance measurement results and the bandgap calculation results are shown in Figure 4 and Figure 5, respectively. To observe absorption due to the film, the transmittance of the quartz substrate was measured as the reference. According to the first-principles calculation, the band structure of Mg(OH)_2_ is direct [31], and thus the band gap can be evaluated by plotting (αhν)^2^ vs. hν, where α is the absorption coefficient and hν is the photon energy. The bandgap for the as-deposited film was found to be around 5.8 eV, which closely matches the reported values [7].

**Figure 6:**
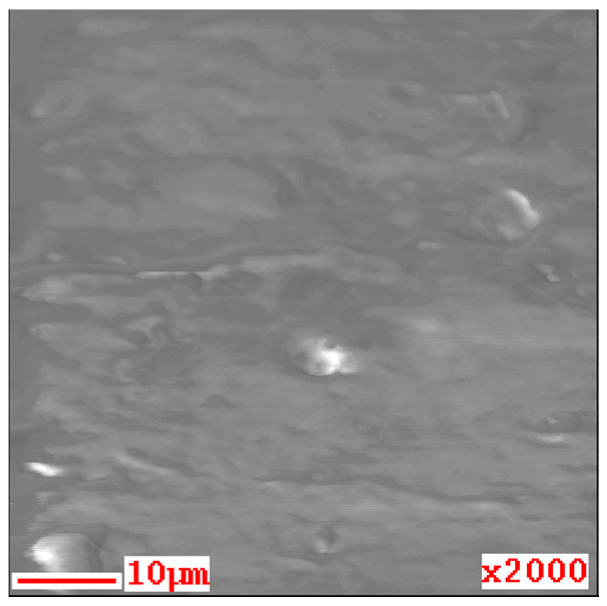
SEM image of the Mg(OH)_2_ film.

**Figure 7:**
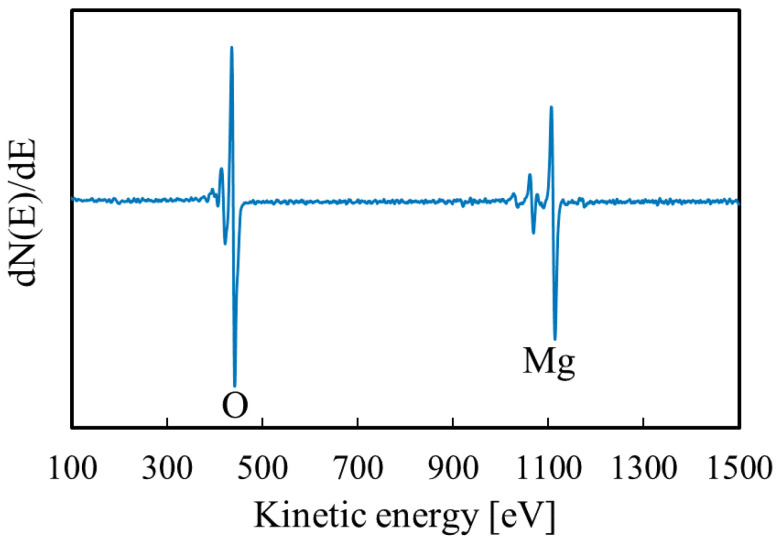
AES spectra of the Mg(OH)_2_ film.

The SEM image of the thin film is shown in Figure 6. Grain images were not observed on the surfaces. In the AES measurement, only the signals of Mg and O were detected, as shown in Figure 7. Thus, by the rinsing process, the contents of the other elements contained in the deposition solution (Na, N) were reduced to below the detection limit of AES (about 1%).

**Figure 8:**
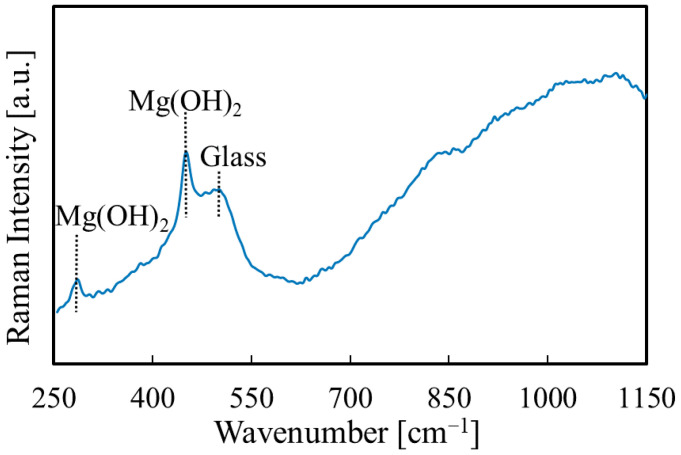
Raman spectrum of the Mg(OH)_2_ film.

**Figure 9:**
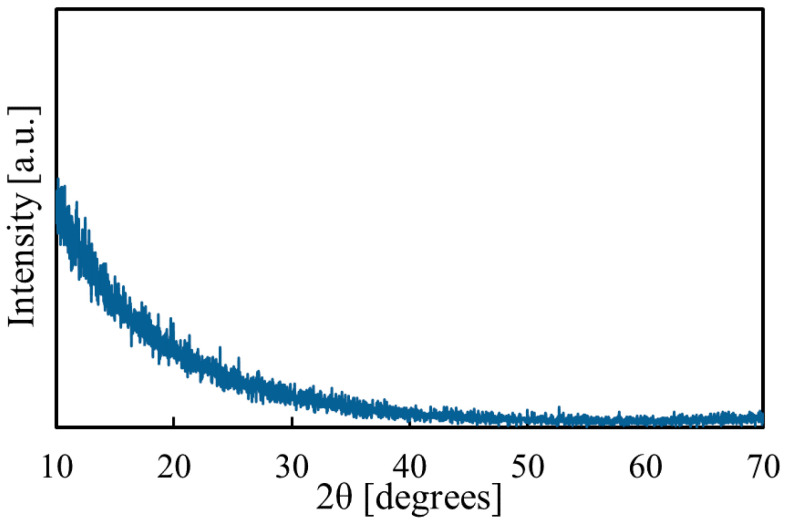
XRD of the Mg(OH)_2_ film.

The Raman measurement results are shown in Figure 8. The peaks of Mg(OH)_2_ and alkali-free glass (substrate) were observed for the thin film [32]. To clarify the structural properties, XRD measurements were also carried out. However, we did not observe any peaks, as shown in Figure 9. Therefore, we think that the films are amorphous.

In the I-V measurement, ohmic behaviors were observed. It was confirmed that alkali-free glass substrates are insulating, and thus the observed current was due to conduction in the film parallel to the surface. Six pairs of electrodes were formed on a sample. The resistivity values calculated from the I-V data were scattered in a range from 4.4 to 8.5 × 10^5^ Ωcm.

### 3.3. Discussion

The I-V results indicate that the films are not insulating but semiconducting. The origin of the conductivity of the Mg(OH)_2_ films is not understood. According to the first-principles calculation, both native defects and impurities can donate carriers in Mg(OH)_2_ [8]. Possible impurities are Na^+^ from NaOH and NO_3_^−^ from Mg(NO_3_)_2_. Although Na and N were not found in the AES measurement, a trace amount of them could be contained in the films and affect the conductivity. In the future, we will attempt intentional impurity doping to control the resistivity. If n- and p-type conductivity of Mg(OH)_2_ is controlled through doping, then Mg(OH)_2_ can be used as a transparent semiconductor to fabricate devices such as diodes and transistors.

Although deposition of dispersions and subsequent drying is a common process for preparing a film, the drying process has not been utilized for fabricating flat, transparent, thin films in electronics. We attempted for the first time to fabricate transparent semiconductor films by DDD in this work. DDD would be advantageous for transparent electronics because films can be deposited at low temperatures using a heater plate only. To deposit a film in a large area by DDD, care must be taken to evenly spread the solution over the entire deposition surface. The solution should be dropped not at a single point but at multiple points.

## 4. Conclusions

In this study, Mg(OH)_2_ thin films were prepared by drop-dry deposition using an aqueous solution containing Mg(NO_3_)_2_ and NaOH, and their elemental composition and optical and electrical properties were evaluated. Optical transmittance in the visible range was 95% or more for the samples with a thickness of about 2 μm. The Raman peaks of Mg(OH)_2_ were observed for the as-deposited film. The bandgap was about 5.8 eV, and the resistivity was of the order of 10^5^ Ωcm.

## Data Availability

The data is contained within the article.
